# Supplementary material for: COZOID: contact zone identifier for visual analysis of protein-protein interactions
Source: BMC Bioinformatics. 2018 Apr 6;19:125. doi: 10.1186/s12859-018-2113-6 (PMC5889581; doi:10.1186/s12859-018-2113-6)
Supplement: Supplementary file 2 — Software build. Executable binary file of the software tool. (ZIP 118,784 kb) [file 12859_2018_2113_MOESM2_ESM.zip › cozoid/java/docs/beansbinding-1.2.1-doc.zip!/org/jdesktop/observablecollections/ObservableCollections.html]

ObservableCollections (Beans Binding)


---


|  |  |  |
| --- | --- | --- |
| **Overview** \| **Package** \| **Class** \| **Use** \| **Tree** \| **Deprecated** \| **Index** \| **Help** | |  |
| PREV CLASS   **NEXT CLASS** | **FRAMES**    **NO FRAMES**     **All Classes** |  |
| SUMMARY: NESTED \| FIELD \| CONSTR \| METHOD | DETAIL: FIELD \| CONSTR \| METHOD |  |


---


## org.jdesktop.observablecollections Class ObservableCollections

```
java.lang.Object
  org.jdesktop.observablecollections.ObservableCollections
```

---

``` public final class ObservableCollections extends java.lang.Object ```

`ObservableCollections` provides factory methods for creating
observable lists and maps.

---

| **Nested Class Summary** | |
| --- | --- |
| `static class` | `ObservableCollections.ObservableListHelper<E>`             `ObservableListHelper` is created by `observableListHelper`, and useful when changes to individual elements of the list can be tracked. |


**Constructor Summary**

`ObservableCollections()`


| **Method Summary** | |
| --- | --- |
| `static <E> ObservableList<E>` | `observableList(java.util.List<E> list)`             Creates and returns an `ObservableList` wrapping the supplied `List`. |
| `static <E> ObservableCollections.ObservableListHelper<E>` | `observableListHelper(java.util.List<E> list)`             Creates and returns an `ObservableListHelper` wrapping the supplied `List`. |
| `static <K,V> ObservableMap<K,V>` | `observableMap(java.util.Map<K,V> map)`             Creates and returns an `ObservableMap` wrapping the supplied `Map`. |

**Methods inherited from class java.lang.Object**

`clone, equals, finalize, getClass, hashCode, notify, notifyAll, toString, wait, wait, wait`

**Constructor Detail**

### ObservableCollections

```
public ObservableCollections()
```


**Method Detail**

### observableMap

```
public static <K,V> ObservableMap<K,V> observableMap(java.util.Map<K,V> map)
```

:   Creates and returns an `ObservableMap` wrapping the supplied
    `Map`.

    :   **Parameters:**: `map` - the `Map` to wrap **Returns:**: an `ObservableMap` **Throws:**: `java.lang.IllegalArgumentException` - if `map` is `null`

---


### observableList

```
public static <E> ObservableList<E> observableList(java.util.List<E> list)
```

:   Creates and returns an `ObservableList` wrapping the supplied
    `List`.

    :   **Parameters:**: `list` - the `List` to wrap **Returns:**: an `ObservableList` **Throws:**: `java.lang.IllegalArgumentException` - if `list` is `null`

---


### observableListHelper

```
public static <E> ObservableCollections.ObservableListHelper<E> observableListHelper(java.util.List<E> list)
```

:   Creates and returns an `ObservableListHelper` wrapping
    the supplied `List`. If you can track changes to the underlying
    list, use this method instead of `observableList()`.

    :   **Parameters:**: `list` - the `List` to wrap **Returns:**: an `ObservableList` **Throws:**: `java.lang.IllegalArgumentException` - if `list` is `null` **See Also:**: `observableList(java.util.List)`


---


|  |  |  |
| --- | --- | --- |
| **Overview** \| **Package** \| **Class** \| **Use** \| **Tree** \| **Deprecated** \| **Index** \| **Help** | |  |
| PREV CLASS   **NEXT CLASS** | **FRAMES**    **NO FRAMES**     **All Classes** |  |
| SUMMARY: NESTED \| FIELD \| CONSTR \| METHOD | DETAIL: FIELD \| CONSTR \| METHOD |  |


---
